# UBE2T: A new molecular regulator of cancer stemness in hepatocellular carcinoma

**DOI:** 10.18632/oncotarget.28033

**Published:** 2021-08-17

**Authors:** Rainbow Wing Hei Leung, Nicole Pui Yu Ho, Carmen Oi Ning Leung, Terence Kin Wah Lee

**Keywords:** beta-catenin, cancer stem cells, UBE2T, hepatocellular carcinoma

Hepatocellular carcinoma (HCC) ranks the 4th leading cause of cancer mortality in the world [1]. Accumulating evidence has emerged in support of the critical role of cancer stem cells (CSCs) to the poor survival of HCC patients as they promote chemoresistance, tumorigenicity and metastasis [2]. Integrative comparative genomic analysis has established molecular similarities between normal liver stem cells and liver CSCs [3], demonstrating the significance of liver CSCs in hepatocarcinogenesis. Therefore, identification of new signaling pathways in regulation of CSCs may improve the prognosis of HCC patients.

To explore for critical molecules/pathways involved in the regulation of liver CSCs, we have performed an integrative analysis of three publicly available datasets involving stemness-related data (GSE5975 and CSCdb) [4, 5] and HCC clinical (liver hepatocellular carcinoma (*TCGA*-LIHC) data. Upon integrative analysis, we showed enrichment of the interstrand cross-link repair pathway, in which ubiquitin conjugating enzyme E2 T (UBE2T) was found to be most upregulated [6]. UBE2T is the E2 ubiquitin-conjugating enzyme of the FA DNA repair pathway, which was first identified in normal hematopoietic stem cells [7]. Clinically, UBE2T was found to be overexpressed in both mRNA and protein levels, and was correlated with poor survival of HCC patients. Using lentiviral-based overexpression and knockdown approaches, UBE2T was found to regulate tumorigencity, self-renewal, tumor invasiveness, and resistance to chemotherapy and molecularly targeted drugs. This result is in line with other studies showing the effect of UBE2T on HCC cell growth *via* regulation of cell cycle related genes [8].

UBE2T was previously reported to regulate protein expression of BRCA1 *via* ubiquitination [9]. Based on the finding of this study, UBE2T exerts its physiological and functional roles through protein-protein interaction. In order to understand the molecular mechanism of how UBE2T regulates CSC properties and tumor behavior, it is crucial to identify its direct interacting partner. By employing tandem affinity purification coupled with mass spectrometry (TAP-MS), we identified for the first time that Mcl-1 ubiquitin ligase E3 (Mule) is the putative protein binding partner of UBE2T. Interestingly, UBE2T regulates Mule protein stability through ubiquitin-proteasomal degradation, and this this effect was dependent on its E2 enzymatic activity at the conserved cysteine residue C86. Clinically, decrease in Mule expression was consistently observed in clinical HCC samples [10]. Specifically, UBE2T induced Mule degradation *via* ubiquitin-mediated degradation at K48. It remains a question whether UBE2T ubiquitinates Mule directly instead of acting as a mediator to transfer ubiquitin to E3 ligases. The direct action of E2 enzymes in the regulation of substrates *via* ubiquitination is not impossible. This piece of exciting data may suggest a new noncanonical role of UBE2T in cancer. Having said that, we cannot exclude the possibility that UBE2T binds to Mule and promote Mule autoubiquitination and degradation. Therefore, further in-depth investigation is required to elucidate the exact mechanism of how UBE2T degrades Mule.

Based on the observation that β-catenin was directly degraded by mule [11], we further hypothesized that UBE2T regulates β-catenin expression *via* degradation of Mule in HCC cells. Firstly, we found a positive correlation between UBE2T and β-catenin protein expression upon alterations of UBE2T expression in HCC cells. Furthermore, UBE2T overexpression augments β-catenin transactivating activity, as evidenced by nuclear accumulation of β-catenin protein, increase in TOP/FOP activity and expression of β-catenin downstream genes. To further confirm that β-catenin is the major effector of UBE2T-mediated CSC functions, we have treated UBE2T repressed HCC cells with CHIR99021, a GSK3β inhibitor. CHIR99021 rescued the suppressive effects of UBE2T inhibition in UBE2T-repressed HCC cells. This *in vitro* data is echoed by the clinical observation that UBE2T was positively correlated with β-catenin in a cohort of HCC samples. Collectively, these results reveal that UBE2T may regulate liver CSC function, at least in part through the β-catenin signaling cascade.

In summary, our study provides a novel prescriptive to understand the physiological and functional roles of UBE2T in cancer. Moreover, our study opens a new therapeutic avenue for targeting HCC by the selective blockade of UBE2T/Mule/β-catenin signaling cascade (Figure 1), warranting a thorough investigation.

**Figure 1:**
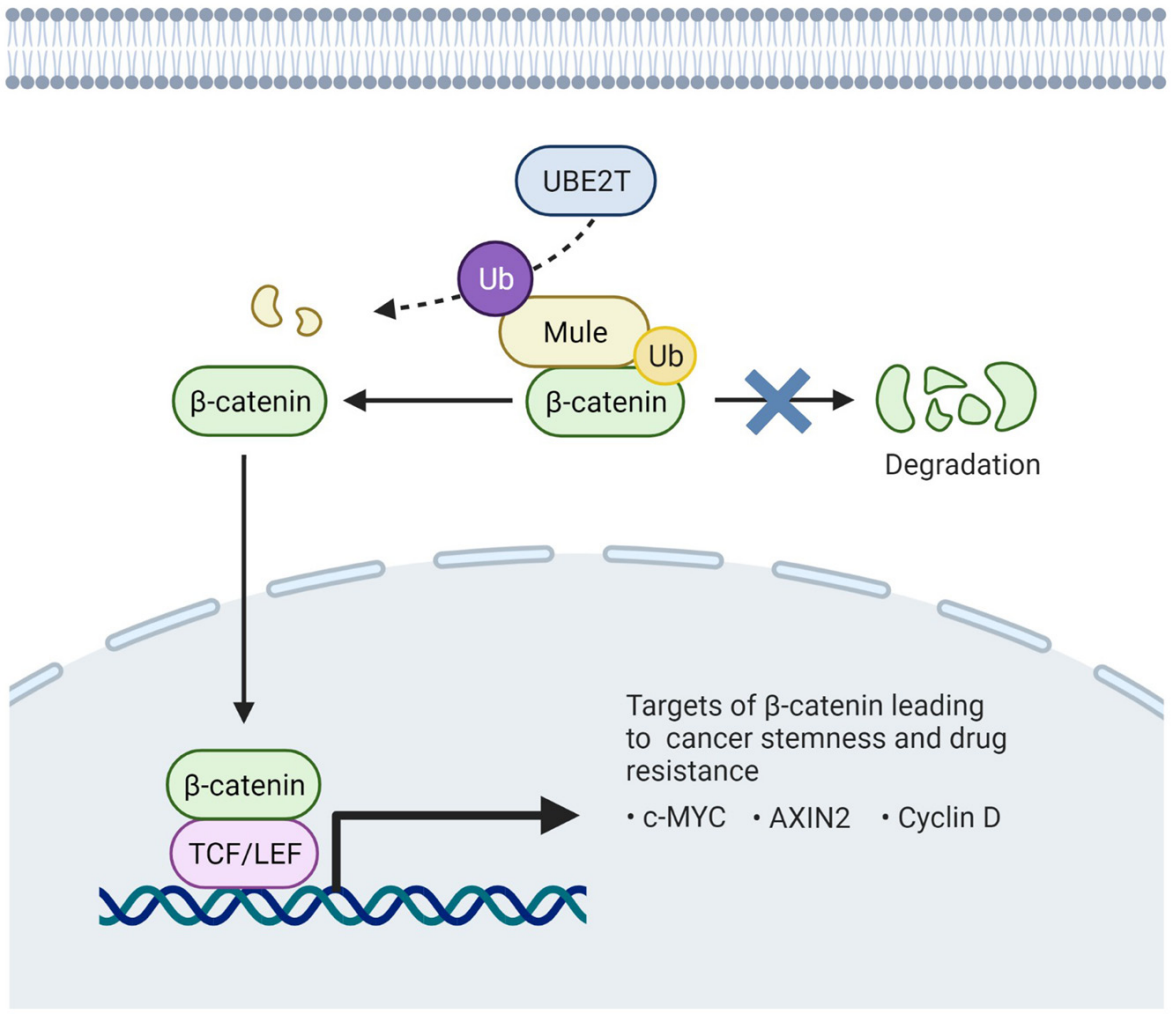
Schematic representation of the interplay of UBE2T and Mule in regulation of β-catenin degradation in HCC.

## Abbreviations

CSCs: cancer stem cells
HCC: hepatocellular carcinoma
Mule: Mcl-1 ubiquitin ligase E3
TAP-MS: tandem affinity purification coupled with mass spectrometry
UBE2T: ubiquitin conjugating enzyme E2 T
